# Inferior Vena Cava Filter Retrieval Trends: A Single-Center Experience

**DOI:** 10.1055/s-0040-1722707

**Published:** 2021-02-10

**Authors:** Filip Ionescu, Nwabundo Anusim, Eva Ma, Lihua Qu, LeAnn M. Blankenship, Michael Stender, Ishmael Jaiyesimi

**Affiliations:** 1 Department of Internal Medicine, Beaumont Health System, Royal Oak, Michigan, United States; 2 Department of Hematology-Oncology, Beaumont Health System, Royal Oak, Michigan, United States; 3 Beaumont Health Research Institute, Royal Oak, Michigan, United States

**Keywords:** IVC filter, retrieval, thrombosis, filter failure, practice trends

## Abstract

Recognition of the adverse events of inferior vena cava filters (VCFs) has prompted the Food and Drug Administration (FDA) to issue safety warnings (2010 and 2014), advocating for removal, once the risk of pulmonary embolism has abated. Despite an initial increase in retrieval rates, these remain low (25–30% at 1 year in 2014). We retrospectively investigated retrieval trends in adults with VCFs placed between 2015 and 2018 at a single institution. The rate of retrievable VCF removal accounting for the competing risk of death was the main outcome. There were 494 VCFs placed (305 retrievable). The cumulative incidence of retrieval remained low (21% at 1 year), even after the second FDA warning (2014). Patients who resumed anticoagulation (AC) at any time were more likely to have retrieval (hazard ratio [HR] = 3.6, *p* < 0.01) and had higher retrieval rates at every time point (31.4 vs. 7.6% at 1 year). Advanced age (HR = 0.98 per year, *p* = 0.004), stroke (HR = 0.28, *p* = 0.028), and active malignancy (HR = 0.42, *p* = 0.006) predicted nonretrieval. Device-related complications were infrequent (<1%) but thrombotic complications occurred early and were more common for nonretrieved VCFs (17 vs. 12%, *p* = 0.29). Revision of guidelines to recommend active surveillance for the ability to tolerate AC in the immediate postimplantation period may improve retrieval rates.

## Introduction

Inferior vena cava filters (VCFs) are devised to mechanically prevent embolization to the pulmonary circulation of thrombi formed in the venous systems of the pelvis and of the lower extremities. The efficacy of VCF in preventing secondary pulmonary embolism (PE) is suggested by many retrospective and observational trials reporting rates of 2 to 4%, 1 2 3 with variable impact in preventing PE-related mortality. 3 4 5 In patients with absolute contraindications to anticoagulation (AC), VCFs represent a reasonable alternative to mitigate the risk of embolization, but they offer no additional benefit in patients who can eventually be transitioned to therapeutic AC and tolerate therapeutic dosing. 6 7 In these cases, VCFs should be removed as soon as possible to prevent device-related complications. Filter utilization has increased in the United States and Europe with the advent of retrievable VCFs, 8 9 raising concerns regarding VCF safety, 10 which have prompted the U.S. Food and Drug Administration (FDA) to issue two device safety warnings in 2010 and 2014, urging physicians to remove filters either, once the risk of pulmonary embolism has abated. 11 12 The recommendations were based on the report of 921 device-related complications over a 5-year period (migration, fracture, and thrombosis). Subsequent studies have reported that the incidence of these complications increases with prolonged use of filters 13 and a decision analysis study found that the risk/benefit profile favors filter removal between 29 and 54 days. 14

Despite an increase in VCF retrieval in the United States following the 2010 FDA advisory statement 15 16 17 18 and several studies reporting retrieval rates of up to 85% following the implementation of structured follow-up programs, 19 20 21 22 23 at a national level, VCF retrieval rates remain low with an estimated average of 25 to 30% at 1 year. 13 16 18 24 25 European retrieval rates tend to be higher but vary from approximately 40 9 26 to 77% at 1 year. 27

Most available U.S. estimates used data prior to the second FDA warning (2014) and were based on national data which are prone to inherent bias (in-hospital data and coding bias) and lacked the granularity to assess the impact of demographic or clinical factors on retrieval rates. Furthermore, recent analyses of the impact of structured VCF follow-up programs have shown these to be effective for increasing retrieval rates. Such a program was not in place at our institution during the study period, and evaluation for filter retrieval was at the discretion of the placing physician. We sought to explore the characteristics of patients receiving VCFs and investigate the contemporary trends of VCF retrieval in a large community hospital in the absence of a standardized follow-up scheme, as well as the factors associated with filter retrieval that may escape analyses based on national data.

## Methods

### Study Design

A single-center, retrospective analysis was conducted at William Beaumont Hospital (Royal Oak, Michigan, United States), a tertiary care center. Patients aged 18 years or older who underwent inferior VCF placement between December 2015 and December 2018 were identified from inpatient records using the International Classification of Diseases, Ninth Revision, Clinical Modification (ICD-9-CM) procedure codes and Current Procedural Terminology Coding System, Fourth Edition (CPT-4) codes. Patients with either permanent (pVCF) or retrievable filters (rVCF) were included. Data recorded from review of electronic medical records included patient demographics (age, sex, and ethnicity), insurance payer, relevant comorbid conditions at the time of the procedure, 17 as well as information regarding VCF type, manufacturer, placing service, indication for placement, and time to VCF retrieval. The indications for filter placement were classified into absolute, relative, and prophylactic as outlined in current guidelines. 28 29 Complications were divided into device-related (filter tilt, filter thrombosis, filter fracture and subsequent embolization, venous wall penetration, etc.) and thrombotic (deep vein thrombosis [DVT] and pulmonary embolism [PE]). Active cancer was defined according to definition of the International Society on Thrombosis and Hemostasis. 30

### Outcomes and Statistical Analysis

The main outcome was the time to inferior VCF retrieval, reported as retrieval rate at 6 months, 1 year, and 2 years. Survival analysis was limited to retrievable filters. Cumulative incidence graphs and estimates were obtained using filter retrieval as event of interest and death as competing risk. Gray's test was used for comparing cumulative incidence curves by variables of interest. Time zero was the time of filter implantation. Patients who did not have filter retrieval by the end of the study period and did not die were censored at the time of last clinical follow-up. A proportional subdistribution hazards regression model with retrieval as the event of interest and death as the competing event was performed to assess the impact of candidate variables on the retrieval rate. Candidate variables were preliminarily tested for significance in a Cox's proportional hazards model using backward and forward regression.

Statistical analysis was performed using R (software version 3.5.0). Categorical variables are described as frequency (percentage). Normal or approximately normal variables are reported using the mean (standard deviation), whereas skewed variables are reported with the median (interquartile range [IQR]). Categorical variables were compared using the Chi-square test or Fisher's exact test. Normal variables were compared using a two-sided Student's *t* -test and ordinal variables used the Kruskal–Wallis test. All *p* -values were two-sided unless otherwise specified, and *p* < 0.05 was considered to indicate statistical significance.

## Results

### Overall Vena Cava Filter Population

**Fig. 1:**
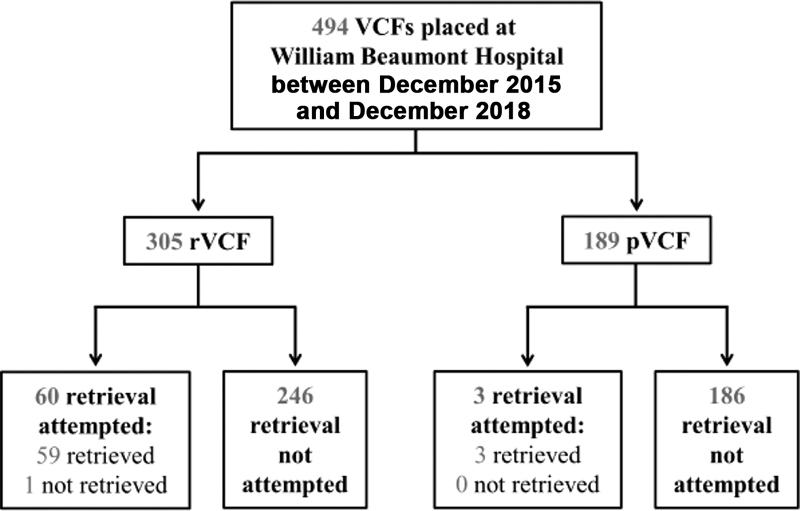
Outline of study population. pVCF, permanent vena cava filter; rVCF, retrievable VCF.

**Fig. 2:**
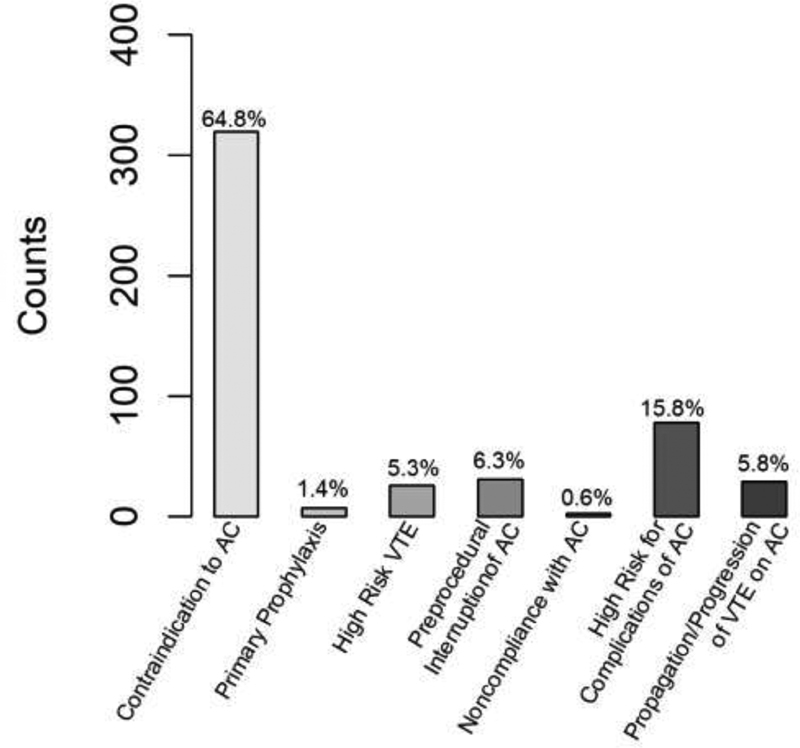
Indications for VCF placement in overall population. AC, anticoagulation; VCF, vena cava filter; VTE, venous thromboembolism.

Over 3 years, 494 VCFs were placed at our institution (305 [62%] were retrievable). Fig. 1 provides an outline of the study population. The majority of the permanent filters were Vena Tech filters (75%), while the retrievable filters were nearly equally represented by Cook Celect (39%), Gunther Tulip (30%), and Option Elite (30%). Baseline characteristics are summarized in Table 1 . Proximal DVTs accounted for the majority of the index VTE events (314, 64%), followed by PE ± DVT (174, 35%); the remainder were placed for prophylactic indications such as trauma, surgical, or medical patients at high risk of developing VTE (6, 1%). Fig. 2 illustrates the indications for VCF placement in the overall population. VCFs were most frequently placed by the interventional radiology service (309, 62%), followed by the vascular surgery (92, 19%) and interventional cardiology services (93, 19%). A total of 162 patients (33%) resumed AC on discharge from the index admission, and up to 239 (48%) were able to resume AC at some point thereafter.

**Table 1:** Demographics and baseline characteristics of VCF population

Demographics	All filters ( *n* = 494)	Retrievable ( *n* = 305)	Permanent ( *n* = 189)	*p* -Value
Age (y)	69 (16)	65 (16)	76 (13)	<0.001
Male sex	249 (50)	168 (55)	81 (43)	0.008
Caucasian ethnicity	332 (67)	207 (68)	125 (66)	0.7
BMI >40 kg/m ^2^	46 (9)	34 (11)	12 (6)	0.07
Medicare/Medicaid	367 (74)	210 (69)	157 (83)	<0.001
Hypertension	370 (75)	217 (71)	153 (81)	0.015
Diabetes	138 (28)	81 (27)	57 (30)	0.4
Coronary artery disease	140 (28)	77 (25)	63 (33)	0.053
Peripheral artery disease	35 (7)	17 (6)	18 (10)	0.1
Heart failure	81 (16)	45 (15)	36 (19)	0.21
Cerebrovascular disease	96 (19)	49 (16)	47 (25)	0.01
CKD ≥grade 3	131 (27)	68 (22)	63 (33)	0.007
Chronic lung disease	88 (18)	42 (14)	46 (24)	0.003
Active malignancy	206 (42)	116 (38)	90 (48)	0.036
IR as placing service	309 (62)	158 (52)	151 (80)	<0.001
Reversible indication a	184 (37)	132 (43)	52 (28)	<0.001
Ever resumed AC	239 (48)	168 (55)	71 (38)	<0.001

Abbreviations: AC, anticoagulation; BMI, body mass index; CKD, chronic kidney disease; IR, interventional radiology; VCF, vena cava filter.

Note: Values represent mean (standard deviation) or *n* (%).

a Reversible indications: bleeding from known and reversible cause other than AC, transient thrombocytopenia, and recent or planned surgery as the only contraindications for AC at time of venous thromboembolism diagnosis.

Seventy (14%) patients suffered from long-term complications associated with VCF and most were thrombotic (68 [14%]: 59 proximal DVTs, 9 PE ± DVT). The cumulative incidences of complications accounting for death as a competing risk were 12% (95% confidence interval [CI]: 9–15%) at 6 months and 14% (95% CI: 10–17%) at 1 year. Among the complications recorded, half occurred within 2.4 months (IQR: 1–8 months) of filter placement and 90% occurred within 1.5 years of postplacement. Device-related complications were rare (total of five [1%]: two VCF thromboses, one filter tilting, one vena cava injury, and one unsuccessful retrieval), and all occurred in the retrievable filter group.

Attempts at filter retrieval were made in 63 cases (13%) and 62 were removed successfully (retrieval success rate 98%).

### Comparison of Retrievable and Permanent Filter Populations

A comparison of baseline characteristics of patients with retrievable and permanent filters is shown in Table 1 . In contrast to patients who received rVCFs, the population for which permanent VCFs were chosen was older, had more comorbid conditions and a perceived poorer prognosis as indicated by more frequent transfer to hospice care within 1 year of filter placement (30 vs. 21% *p* = 0.02) and diagnoses of active malignancy (48 vs. 38%, *p* = 0.04). More rVCF patients were able to resume AC compared with pVCF patients both at discharge from the index admission (39 vs. 23%, *p* < 0.001) and at any time thereafter (55 vs. 38%, *p* < 0.001). More rVCF were placed for reversible indications (43 vs. 28%, *p* < 0.001). Complications were more frequent among rVCFs (17 vs. 11%). Notably, one PE and one DVT occurred at 1 month and 1 week, respectively, after filter retrieval.

### Retrievable Vena Cava Filters

Of the 305 patients with rVCFs, a total of 60 filter retrieval attempts were made and 59 (19%) were successful ( Fig. 1 ). Baseline characteristics and comorbid conditions of the rVCF population are shown in Table 2 . Interventional radiology remained the most frequent service placing both retrieved (58%) and nonretrieved filters (50%). Absolute indications were predominant (215, 70%), with percentages in the relative indication (85, 28%) and prophylactic indication (5, 2%) categories comparable to those observed in the overall VCF population; this distribution did not differ significantly between retrieved and nonretrieved filters ( *p* = 0.95). The primary VTE event that prompted filter placement and a comparison of specific indications between retrieved and nonretrieved rVCFs are shown in Table 3 .

**Table 2:** Demographics and baseline characteristics of patients with rVCFs

Demographics	Retrieved ( *n* = 59)	Not retrieved ( *n* = 246)	*p* -Value
Age (y)	57 (15)	67 (16)	<0.001
Male sex	33 (56)	135 (55)	0.9
Caucasian ethnicity	40 (68)	167 (68)	1
BMI >40 kg/m ^2^	9 (15)	25 (10)	0.26
Medicare/Medicaid	32 (54)	149 (83)	0.007
Hypertension	36 (61)	181 (74)	0.056
Diabetes	11 (19)	70 (29)	0.12
Coronary artery disease	8 (14)	69 (28)	0.02
Peripheral artery disease	0 (0)	17 (7)	0.051
Heart failure	1 (2)	44 (18)	<0.001
Cerebrovascular disease	3 (5)	46 (19)	0.009
CKD ≥grade 3	11 (19)	57 (23)	0.45
Chronic lung disease	2 (3)	40 (16)	0.01
Active malignancy	12 (20)	104 (42)	0.002

Abbreviations: BMI, body mass index; CKD, chronic kidney disease; rVCF, retrievable vena cava filter.

Note: Values represent mean (standard deviation) or *n* (%).

**Table 3:** Indications and complications of retrieved and nonretrieved rVCFs

	All retrievable ( *n* = 305)	Retrieved ( *n* = 59)	Not retrieved ( *n* = 246)	*p* -Value
IR as placing service	158 (22)	34 (58)	124 (50)	0.31
Primary VTE event				0.13 b
DVT	191 (63)	31 (52)	160 (65)	
PE ± DVT	110 (36)	27 (46)	83 (34)	
Prophylactic	4 (1)	1 (2)	3 (1)	
Indication for IVC filter				**<0.001** ^b^
Contraindication to AC	200 (65)	39 (66)	161 (65)	
Propagation/progression of VTE on AC	15 (5)	2 (3)	13 (5)	
High risk for complications of AC	38 (12)	0 (0)	38 (15)	
Preprocedural interruption of AC	25 (8)	8 (14)	17 (7)	
Noncompliance with AC	2 (1)	0 (0)	2 (1)	
High risk VTE	20 (7)	9 (15)	11 (4)	
Primary prophylaxis	5 (2)	1 (2)	4 (2)	
Reversible indication a	210 (69)	37 (63)	95 (39)	<0.001
Ever resumed AC	168 (55)	49 (83)	119 (48)	<0.001
All complications	50 (16)	7 (12)	43 (17)	0.29
Thrombotic complications	49 (16)	6 (10)	43 (17)	0.17
Death or hospice within 1 year	98 (32)	1 (2)	97 (39)	<0.001

Abbreviations: AC, anticoagulation; DVT, deep vein thrombosis; IR, interventional radiology; IVC, inferior vena cava; PE, pulmonary embolism; rVCF, retrievable vena cava filter; VTE, venous thromboembolism.

Note: Values represent *n* (%).

a Reversible reasons: bleeding from known and reversible cause other than AC, transient thrombocytopenia and recent or planned surgery as the only contraindications for AC at the time of VTE diagnosis.

b *p* -Value for the association.

Half of all complications were observed within 3 months (IQR: 1–10 months) and 90% in the first 1.5 years of postplacement. Retrieved rVCFs were associated with fewer complications overall (12 vs. 17%) and fewer thrombotic complications (10 vs. 17%); the difference was primarily driven by DVTs. All filter-related complications (vena cava injury, filter tilting, filter thrombosis, and unsuccessful retrieval) occurred in the nonretrieved rVCF population. Even in patients who resumed AC, nonretrieved rVCF patients had the higher rate of overall complications (25 vs. 12%, *p* = 0.06).

By the time of discharge from the index admission, 119 (39%) patients with rVCFs could be restarted on AC, and an additional 16% (total 168, 55%) were able to restart AC at some point after discharge.

**Fig. 3:**
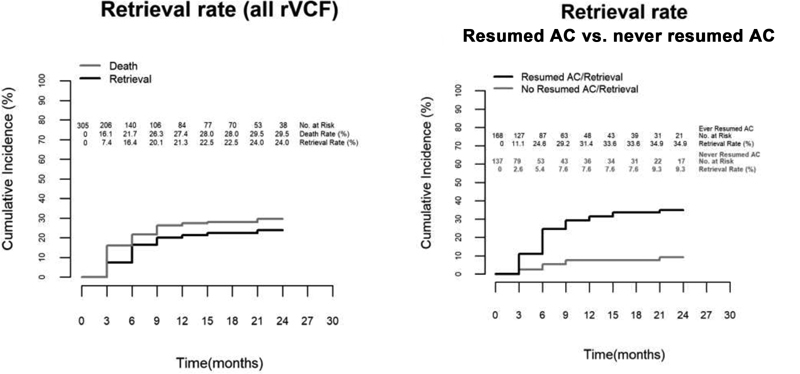
( **A** ) Cumulative incidence of VCF retrieval over 2-year follow-up accounting for death as a competing risk in overall retrievable filter population. VCF, vena cava filter; VTE, venous thromboembolism. ( **B** ) Cumulative incidence of VCF retrieval over 2-year follow-up accounting for death as a competing risk in retrievable filter population who ever resumed AC versus who never resumed AC. AC, anticoagulation; rVCF, retrievable vena cava filter; VTE, venous thromboembolism.

The cumulative incidence plotting VCF retrieval as the event of interest and death as a competing risk is shown in Fig. 3A . The overall retrieval rates for rVCFs were 16% at 6 months, 21% at 1 year, and 24% at 2 years. Among removed filters, the median time to device retrieval was 4 months (IQR: 2–6 months). The cumulative incidence of VCF retrieval accounting for the competing risk of death in patients who ever resumed AC versus those who did not is shown in Fig. 3B . The former group exhibited significantly higher retrieval rates at every time point (Gray's test *p* < 0.001), with retrieval rates exceeding 30% at 1 year. By contrast, being able to resume AC at discharge did not exhibit such an association ( *p* = 0.43). Other demographics, such as sex or ethnicity, critical illness defined as admission to intensive care unit during the index hospitalization, the index thrombotic event, the placing service, or having a reversible indication for AC did not show a significant relation with retrieval in univariate analysis.

The final model identified resumption of AC at any time (HR = 3.6, 95% CI: 1.8–6.9, *p* < 0.001) as a predictor of retrieval after adjusting for age, CVD and active malignancy. Increasing age (HR = 0.98 per 1-year increase, 95% CI: 0.96–0.99, *p* = 0.004), a diagnosis of cerebrovascular disease (HR = 0.28, 95% CI: 0.09–0.9, *p* = 0.002), or active malignancy (HR = 0.42, 95% CI: 0.2–0.8, *p* = 0.006) was independently associated with a lower likelihood of retrieval.

## Discussion

The current study uses single-institution data to investigate the trends in VCF placement and retrieval following the second FDA safety warning. Our main findings are as follows: (1) in the absence of structured follow-up program, the retrieval rate of rVCFs remained low (21% at 1 year) and below the national average (25–30% at 1 year), even after the second FDA warning in 2014; (2) the ability to resume AC at any time following filter placement was identified as the only predictor of retrieval; (3) filter removal had a high success rate (98%); and (4) long-term complications were common (14% of overall VCF population).

In our population, most VCFs were retrievable (62%). It is worthwhile to mention that a sizeable proportion were represented by older generations of filters which have anecdotally been associated with a lower rate of complications and higher retrieval success rates (although the latter was close to 100% in our cohort). Consistent with other reports, 31 we observed a preference for implanting permanent filters in an older and sicker population. These patients also had a perceived poorer long-term prognosis with more frequent diagnoses of active malignancy and transitions to hospice within 1 year of VCF placement. This finding, along with the observation that perceived reversible indications prompted placement of retrievable filters more frequently, suggests that general clinical impression plays a significant role in selection of filter type. Similar to published data, 31 we recorded marginally fewer complications in permanent filter patients when compared with retrievable filter patients.

Several studies have reported decrements in VCF use following the publication of the 2010 and 2014 FDA safety advisories, 15 32 33 but very few authors found an increase in the retrieval rate. A 2017 study by Brown et al, 18 using national data, found that rates had increased slightly from roughly every one in seven VCFs being retrieved at 1 year in 2010 to up to one in four retrieved in 2014. Another study using Medicare claims data reported a 22.1% retrieval rate per annual filters placed in 2016. 16 In our cohort, the overall retrieval rate of 21% at 1 year was comparable to the 2014 estimate. When attempted, the success rate of filter retrieval was high at 98%, similar to other reports. 34 Notably, retrievals were significantly more frequent for patients who were able to resume AC (31.4 vs. 7.6% at 1 year) and reinitiation of AC was a strong predictor of retrieval (HR = 3.6), even when accounting for advanced age and comorbid conditions. By contrast, Brown et al found only a weak correlation between time to AC and time to retrieval. This observation suggests that, at an institutional level, AC is generally acknowledged as the preferred therapeutic modality for VTE and that complications of prolonged filter dwell time are increasingly recognized. The clinical impression that a particularly frail patient population will be unable to resume AC and may benefit from a converting the initial status of a VCF from temporary to permanent is a variable that could not be included in our models and that may also account for the low retrieval rate in our cohort. However, even when AC was resumed which is a fair surrogate of the temporary intent for the VCF, the retrieval rate remained suboptimal as patients who had nonretrieved filters exhibited a higher frequency of complications (25 vs. 10%). 35 36 37

An interesting finding was that the ability to restart AC upon discharge from the index admission was not significantly associated with retrieval; the lack of statistical significance may owe to the smaller sample size (only ∼40% resumed AC at discharge). In contrast, the percentage of patients who were able to tolerate AC following discharge increased to greater than half (55%), suggesting that active reevaluation for the ability to tolerate therapeutic AC or to check for resolution of conditions placing the patient at high risk for VTE is important for increasing retrieval rates. Others have found a substantial increase in retrieval rates (up to 90%) when certain follow-up strategies were implemented after filter placement. 19 20 21 22 23 These include use of multidisciplinary teams, standardized protocols, patient education, automated reminder, or referral systems and creation of dedicated VCF clinics. Such a standardized, structured follow-up program was not utilized at our institution during the study period, and despite limited data showing efficacy in increasing filter retrieval, a specific recommendation is not present in current guidelines. As of 2019, a dedicated VCF clinic was implemented as part of the Interventional Radiology Department for surveillance of VCFs placed in our institution. The authors plan to evaluate the impact of this intervention as a subsequent analysis.

Despite recent concerns regarding device-related complications, these were infrequent in our cohort (<1%). The addition of thrombotic events raised the rate of complications to 14% of all VCFs (most were proximal DVTs). Nonretrieval was associated with a higher rate of complications (17 vs. 11%) and these occurred early in the postinterntional period, with half diagnosed within 2 to 3 months of filter placement. With a median time to filter removal of 4 months (IQR: 2–6 months), most VCFs are retrieved too late to prevent complications. Furthermore, longer dwell times are associated with a lower retrieval success rate. 38 39 Early retrieval within 30 to 60 days of postplacement, as supported by others, 14 is an optimal strategy. An interesting observation, which was recently corroborated by others, 40 was that AC did not impact the rate of complications, suggesting that AC is not protective against thrombosis in patients with indwelling VCFs and retrieval should be prioritized.

## Limitations and Strengths

This retrospective observational study has several inherent limitations, such as hidden confounders and selection bias. Numerous variables that would have merited inclusion in the analyses were unavailable, such as information about symptoms of VCF complications, the exact time of reinitiation of AC or the exact indication for filter placement. Importantly, information regarding the initial intent for filter placement (temporary vs. permanent) was unavailable for most patients. Many patients were classified as inadequate candidates for AC as a consequence of the subjective impression of the prescribing physician. Furthermore, given the retrospective nature of the analysis, all recorded filter-associated complications were discovered due to the emergence of symptoms or incidentally on imaging obtained for a different indication; asymptomatic complications, such as subclinical thrombosis, vena cava injury, or filter tilting, may have been missed. In addition, some complications or retrievals may have been addressed in different hospital systems and obscured during our analysis. Another important limitation is our inclusion of patients with filters implanted in 2018, who would have had limited follow-up at the time of analysis which likely led to underestimation of cumulative retrieval rates at 1 year.

Despite these limitations, the study offers new data on inferior VCF placement and retrieval from a single institution perspective, and it addresses several shortcomings that are intrinsic to prior population-based analyses, such as the availability of detailed clinical data. By design, it can only report associations and cannot investigate causality. Prospective studies which further investigate strategies to improve patient and device selection and to increase device retrieval are warranted.

## Conclusion

Despite efforts to increase awareness of inferior VCF-related complications, the retrieval rate remained low (21% at 1 year) and apparently failed to increase compared with rates reported in 2014 before the second FDA warning. Nonretrieval filters increased the risk of thrombotic events and may have provided false reassurance that AC is not required; thrombotic complications, however, were frequent, occurred early, and concomitant AC did not appear to mitigate the risk. With a very high removal success rate, early filter retrieval emerges as the best preventive strategy once patients are able to safely resume AC and undergo the procedure. Resumption of AC proved the strongest independent predictor of retrieval in our population. Our findings strengthen those of prior studies that have reported on the efficacy of active long-term surveillance strategies for increasing retrieval rates. Current guidelines should be revised to recommend this approach.
